# Armored CAR T cells enhance antitumor efficacy and overcome the tumor microenvironment

**DOI:** 10.1038/s41598-017-10940-8

**Published:** 2017-09-05

**Authors:** Oladapo O. Yeku, Terence J. Purdon, Mythili Koneru, David Spriggs, Renier J. Brentjens

**Affiliations:** Department of Medicine, Memorial Sloan Kettering Cancer Center, New York, New York, 10065 USA

## Abstract

Chimeric antigen receptor (CAR) T cell therapy has shown limited efficacy for the management of solid tumor malignancies. In ovarian cancer, this is in part due to an immunosuppressive cytokine and cellular tumor microenvironment which suppresses adoptively transferred T cells. We engineered an armored CAR T cell capable of constitutive secretion of IL-12, and delineate the mechanisms via which these CAR T cells overcome a hostile tumor microenvironment. In this report, we demonstrate enhanced proliferation, decreased apoptosis and increased cytotoxicity in the presence of immunosuppressive ascites. *In vivo*, we show enhanced expansion and CAR T cell antitumor efficacy, culminating in improvement in survival in a syngeneic model of ovarian peritoneal carcinomatosis. Armored CAR T cells mediated depletion of tumor associated macrophages and resisted endogenous PD-L1-induced inhibition. These findings highlight the role of the inhibitory microenvironment and how CAR T cells can be further engineered to maintain efficacy.

## Introduction

Adoptive transfer of chimeric antigen receptor (CAR) T cells is an exciting form of immunotherapy that has garnered interest for the treatment of solid tumor malignancies in recent years. Successes in the management of hematologic malignancies such as CD19+ B-cell Acute lymphoblastic leukemia (ALL) has led to investigation of this modality for the treatment of solid tumors^1^. However, to-date, clinical responses in solid tumors have been more modest^2–6^.

Despite initially effective cytotoxic therapy for ovarian cancer, the mortality rate for this disease remains high. This is due in large measure to the eventual development of multidrug resistant disease in the relapsed/ platinum-refractory setting. As such, there remains a need for new therapeutic modalities. Prior efforts at CAR T cell therapy for ovarian cancer using single chain variable fragments (scFv) directed against the folate receptor were largely unsuccessful in clinical trials^5^ despite promising preclinical data^7, 8^. It has been suggested that poor trafficking, limited persistence and T-cell inhibitory activity in patients’ serum contributed to the observed lack of efficacy^5^. This highlights the contribution of the tumor microenvironment to the failure of adoptively transferred T cell therapy.

The tumor microenvironment in ovarian cancer is not only composed of inhibitory cellular elements, but also an immunosuppressive ascitic microenvironment in patients with peritoneal disease^9^. In both patients and murine models, immunosuppressive cytokines such as IL-4, IL-6, LIF, IL-10 and TGF-β have also been reported^10, 11^. The inhibitory cellular microenvironment has been reported to consist of myeloid derived suppressor cells (MDSCs), tumor associated macrophages (TAMs) and regulatory T cells^12^ that potentially mediate suppression of T cells^13–15^. Finally, elaboration of immunosuppressive ligands by the tumor, stromal cells or tumor associated cellular elements have also been described. A prominent example of this is the expression of programed death ligand 1 (PD-L1) which can be upregulated by ovarian cancer cells^16^ and macrophages^17, 18^, and has been shown to confer poor outcomes in some studies^19, 20^. For CAR T cell therapy to be effective for the management of ovarian cancer, the cumulative supressive effects of the microenvironment must be overcome.

Improvements have been made to the core design of CARs in an attempt improve the efficacy of CAR T cells^21^. These enhancements include engineering of 1 or 2 additional costimulatory molecules in addition to the CD3ζ signaling domain. These “second generation” and “third generation” CARs respectively, have yielded incremental improvements in activation, proliferation and cytotoxicity in various preclinical tumor models^22–24^, but none of them have translated to gains in clinical trials for solid tumor malignancies. Further genetic modifications to second generation CAR constructs with pro-inflammatory cytokines or ligands have also been described^25^. These “armored” CAR T cells have been further engineered to secrete cytokines or express soluble or tethered ligands designed to further improve CAR T cell efficacy^25^. One promising candidate is interleukin-12 (IL-12), a pro-inflammatory cytokine recognized for its ability to enhance the cytotoxic capability of CD8+ cells^26^, mitigate antigen-loss tumor escape via recruitment and engagement of macrophages^27^, enhance antigen cross presentation and reprogram MDSC’s^28, 29^. For this reason, exogenous IL-12 therapy has been explored for the treatment of several solid tumor malignancies^30–33^. Treatment with intraperitoneal (i.p.) IL-12 for ovarian cancer resulted in stable disease as the reported best response^33^. However, serious toxicities have been described in both preclinical models and clinical applications of IL-12^33–35^.

In separate previous reports, we have described the efficacy of second generation^36^ and IL-12 secreting armored^37^ CAR T cells directed against the retained portion of Muc-16 (Muc16^ecto^) on human ovarian cancer cells. In SCID-Beige mice, elaboration of IL-12 significantly augmented CAR T-cell efficacy *in vitro* and led to eradication of disseminated disease in a portion of treated mice^37^. In this report, we extend our prior work via utilization of a syngeneic model of murine ovarian peritoneal carcinomatosis to characterize the mechanisms of efficacy of IL-12 secreting CAR T cells. Herein we show that IL-12 armored CAR T cells overcome the inhibitory ascitic microenvironment, alter the ascitic cytokine and TAM microenvironment, and overcome PD-L1-mediated inhibition. Finally, we also present pharmacotoxicity data supporting the safety of IL-12 secreting CARs.

## Results

### 4H1128ζ-IL12 T cells secrete more inflammatory cytokines and show superior cytotoxicity *in vitro*

**Figure 1:**
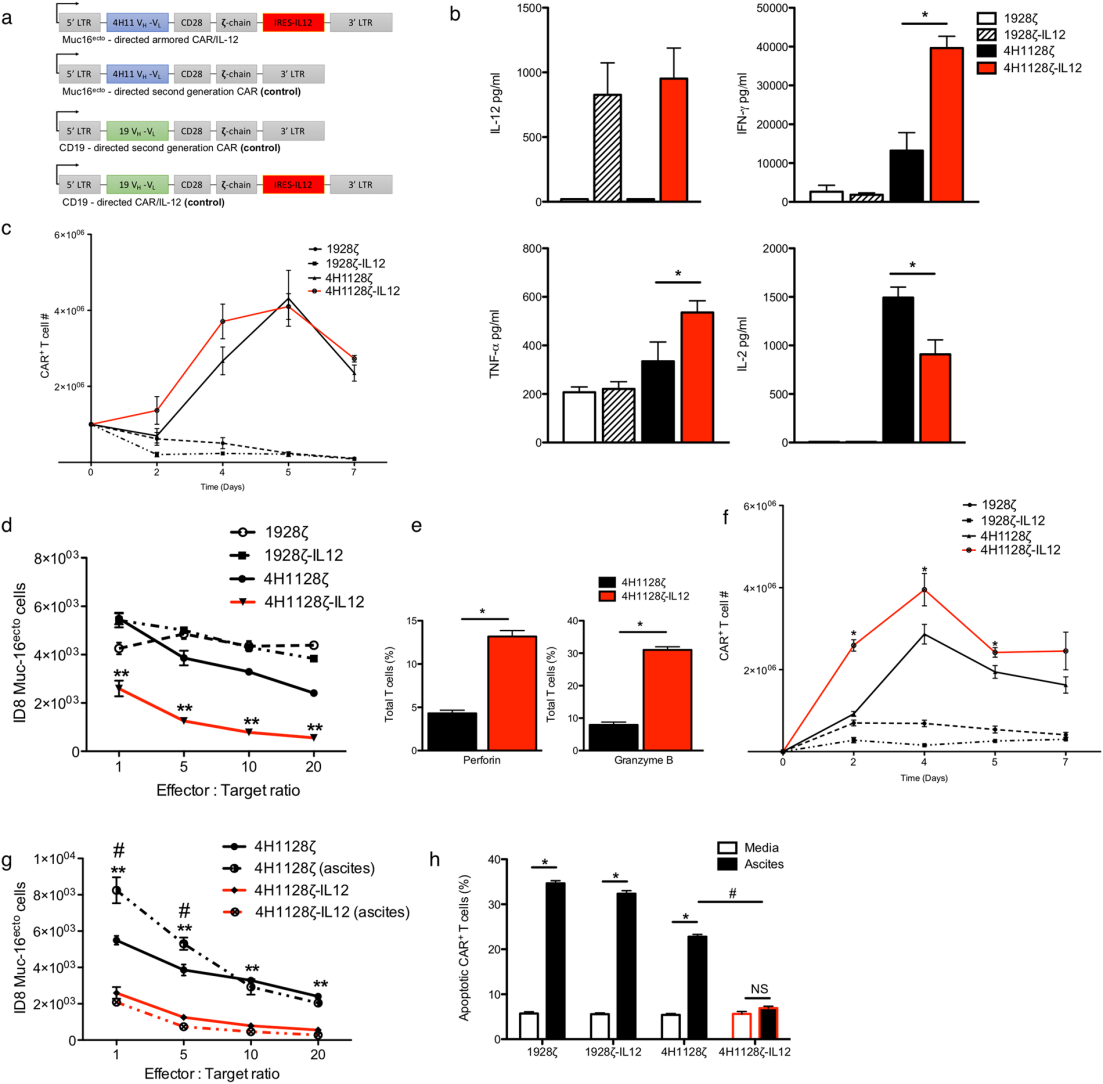
Muc16^ecto^ specific CAR T cells modified to secrete IL-12 elaborate more inflammatory cytokines, exhibit improved proliferation, increased cytotoxicity and decreased apoptosis *in vitro*. (**a**) Schematic representations of Muc16^ecto^- directed second generation CAR (4H1128ζ) modified to express murine IL-12 following an IRES element (4H1128ζ-IL12), and irrelevant CD19- directed second generation (1928ζ) and IL-12 modified (1928ζ-IL12) CARs. (**b**) *In vitro* cytokine analysis of supernatants obtained from coculture of indicated CAR T cells with ID8-Muc16^ecto^ cells for 16 hr. IFN-γ: 4H1128ζ-IL12 vs 4H1128ζ, *p = 0.003. TNF-α: 4H1128ζ-IL12 vs 4H1128ζ CAR T cells, *p = 0.012. IL-2: 4H1128ζ-IL12 vs 4H1128ζ, *p = 0.045. Data are plotted as mean ± SEM (**c**). CAR T cell proliferation assay with indicated CAR T cells cocultured with ID8-Muc16^ecto^ cells. (**d**) *In vitro* cytotoxicity assay of indicated CARs cocultured with ID8-Muc16^ecto^ for 16 hr at the indicated effector: target ratios (E:T) on the x-axis, **p < 0.001. (**e**) Expression levels of perforin and granzyme B in 4H1128ζ-IL12 vs 4H1128ζ CAR T cells, *p < 0.0001 (**f**). CAR T cell proliferation assay with indicated CAR T cells cocultured with ID8-Muc16^ecto^ cells in the presence of cell-free pooled ascites. 24 hr (*p < 0.001), 48 hr (*p = 0.046), 5 days (*p = 0.039). (**g**) *In vitro* cytotoxicity assay of indicated CARs cocultured with ID8-Muc16^ecto^ for 16 hr in the presence of cell-free pooled ascites. 4H1128ζ-IL12 vs 4H1128ζ CAR T cells in ascites (*p < 0.01). 4H1128ζ vs 4H1128ζ ascites (^#^p < 0.01). (**h**) Indicated CAR T cells cocultured with ID8-Muc16^ecto^ cells for 48 hr in the presence of complete media or ascites. Cells were gated on CAR T+ cells prior to gating on annexin V/DAPI. *p < 0.01, ^#^p < 0.01. Data are plotted as mean ± SEM. Data shown are pooled results from 3 independent experiments. Statistics performed using unpaired two-sided T test.

Schematic representations of the retroviral chimeric antigen receptor (CAR) constructs used in this study are shown in Fig. 1a. We generated both second generation Muc16^ecto^ -directed (4H1128ζ) and irrelevant CD19-directed (1928ζ) CAR constructs based on our previously published SFG retroviral vector^36, 38^. To generate the murine IL-12-secreting armored constructs, we further modified both 4H1128ζ and 1928ζ with an IRES element upstream of the murine IL-12 α and β subunit fusion (flexi-IL-12)^38^ to generate 4H1128ζ-IL12 and 1928ζ-IL12 respectively. Functionality of the armored constructs was measured by soluble IL-12 production via coculture of transduced T cells with ID8 cells expressing Muc16^ecto^ (ID8-Muc16^ecto^) for 16 hr and results are shown in Fig. 1b. Constitutive mouse IL-12 production was measurable in both 4H1128ζ-IL12 and 1928ζ-IL12 CAR T cells (Fig. 1b). Next, we assessed the production of IL-2, IFN-γ and TNF-α (Fig. 1b). 4H1128ζ-IL12 T-cells secreted significantly increased IFN-γ (*p = 0.003) and TNF-α (*p = 0.045) compared to 4H1128ζ T cells and control CD19-directed T cells (1928ζ, 1928ζ-IL12). In the absence of relevant antigen stimulation, constitutive IL-12 produced by 1928ζ-IL-12 did not lead to increased production of IL-2, IFN-γ or TNF-α. We found decreased IL-2 production by 4H1128ζ-IL12 T cells compared to 4H1128ζ (*p = 0.012), consistent with prior reports showing sensitization of T cells to IL-2 in the presence of IL-12^38, 39^. IL-12 has also been shown to drive T cells towards a terminally differentiated effector phenotype, thus providing another potential explanation for decreased IL-2 production^40^. We did not observe any differences in proliferation between 4H1128ζ T cells and 4H1128ζ-IL12 T cells in complete media (Fig. 1c). To characterize the cytotoxic efficacy of 4H1128ζ-IL12 T cells, we performed a 16 hr coculture assay with ID8-Muc16^ecto^ cells (Fig. 1d). 4H1128ζ-IL12 T cells were significantly more effective at killing tumor cells compared to 4H1128ζ T cells at all effector to target ratios (**p < 0.001). The increase in cytotoxic efficacy was consistent with increased expression of intracellular perforin and granzyme B in 4H1128ζ-IL12 T cells compared to 4H1128ζ T cells (Fig. 1e) (*p < 0.0001).

### 4H1128ζ-IL12 T cells proliferate better, retain cytotoxicity and resist apoptosis in the ascites microenvironment

The asities microenvironment is generally considered to be immunosuppressive and has been shown to contain high levels of immunosuppressive cytokines such as IL-10 and IL-6^41, 42^ which could inhibit T cell function. We assessed CAR T-cell proliferation in the presence of cell-free pooled ascites derived from tumor-bearing mice (Fig. 1f). As shown in Fig. 1f, 4H1128ζ CAR T cells did not proliferate as robustly as 4H1128ζ-IL12 CAR T cells at 24 hr (*p < 0.001), 48 hr (*p = 0.046) and 120 hr (Day 5, *p = 0.039) after coculture with ID8-Muc16^ecto^ (Fig. 1f). Furthermore, proliferation of 4H1128ζ T cells was blunted between 24 hr and 48 hr (Fig. 1f). To be efficacious in ascites, the predominant ovarian cancer tumor microenvironment, CAR T cells not only need to expand but also need to retain cytotoxic capability. Similar to conditions in complete media, 4H1128ζ-IL12 T cells were more cytotoxic compared to 4H1128ζ T cells in the presence of cell-free pooled ascites (*p < 0.01). Comparison of cytotoxicity between 4H1128ζ and 4H1128ζ-IL12 T cells in the presence of media and ascites demonstrated statistically significant diminution in the cytotoxic capacity of 4H1128ζ T cells (^#^p < 0.01) in the presence of ascites (Fig. 1g). There were no significant differences in the efficacy of 4H1128ζ-IL12 T cells in the presence of ascites compared to complete media (Fig. 1g). Ascites has been shown to be toxic to T cells^43^. We evaluated the role of ascites in suppressing expansion of adoptively transferred T cells via induction of apoptosis. 1928ζ, 1928ζ-IL12, 4H1128ζ and 4H1128ζ-IL12 T cells were cocultured with ID8-Muc16^ecto^ cells in the presence of complete media or ascites and stained with annexin V after 48 hr (Fig. 1h). Apoptotic rates among CD3^+^ CAR^+^ T cells were similar at 48 hr in complete media. However, in the presence of ascites, 1928ζ (*p < 0.05), 1928ζ-IL12 (*p < 0.05) and 4H1128ζ T cells (*p < 0.01) exhibited significantly higher apoptotic percentages compared to 4H1128ζ-IL12 T cells. Apoptosis was not significantly increased in 4H1128ζ-IL12 T cells cocultured with tumor cells in the presence of complete media compared to ascites (N.S) and there were significantly less apoptotic 4H1128ζ-IL12 T cells compared to 4H1128ζ T cells when both were cultured in ascites (^#^p < 0.01). Taken together, secretion of IL-12 promotes increased proliferation, cytotoxicity and confers resistance to apoptosis in T cells activated in the presence of an inhibitory ascites microenvironment.

### 4H1128ζ-IL12 T cells promote survival in an advanced syngeneic model of peritoneal carcinomatosis in an autocrine-dependent manner

**Figure 2:**
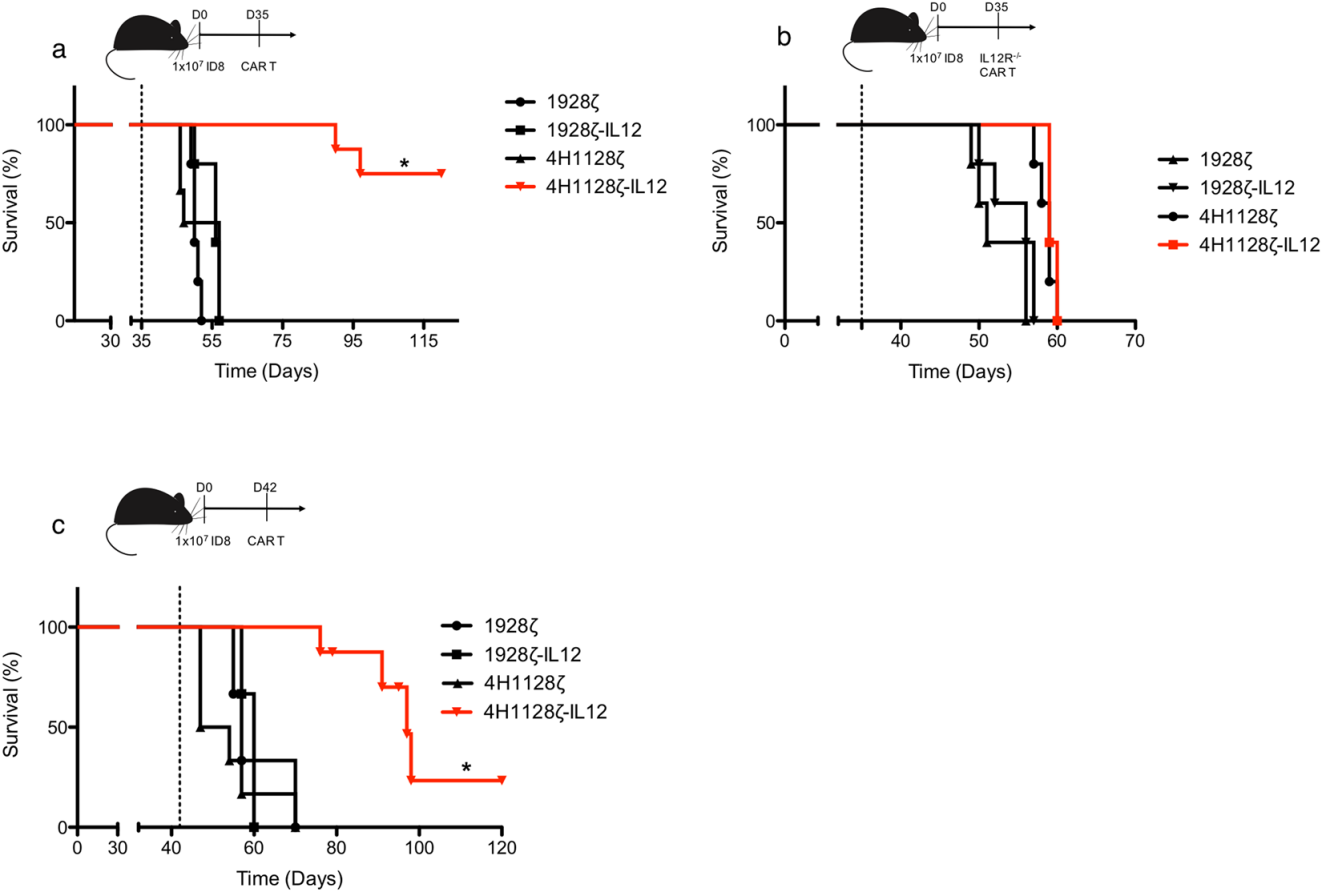
IL-12-secreting CAR T cells prolong survival in an advanced tumor model of ovarian peritoneal carcinomatosis and is dependent on autocrine IL-12 signaling. (**a**) Female wild type C57BL/6 mice (WT) between 6–8 weeks old were inoculated with 1 × 10^7^ ID8-Muc16^ecto^ cells i.p and treated 35 days later with 2 × 10^6^ indicated CAR T cells i.p., *p = 0.02. Number of mice per group: 5 (1928ζ), 5 (1928ζ-IL12), 6 (4H1128ζ), 8 (4H1128ζ-IL12). (**b**) Female WT mice between 6–8 weeks old were inoculated with 1 × 10^7^ ID8-Muc16^ecto^ cells i.p and treated 35 days later with 2 × 10^6^ CAR T cells (i.p) derived from IL-12R^−/−^ splenocytes. Number of mice per group: 5 (1928ζ), 5 (1928ζ-IL12), 5 (4H1128ζ), 5 (4H1128ζ-IL12). (**c**) Female WT mice between 6–8 weeks old were inoculated with 1 × 10^7^ ID8-Muc16^ecto^ cells i.p and treated 42 days later with 2 × 10^6^ indicated CAR T cells i.p, *p < 0.01. Number of mice per group: 5 (1928ζ), 5 (1928ζ-IL12), 6 (4H1128ζ), 8 (4H1128ζ-IL12). Results are pooled from 2 independent experiments. Statistical analysis performed using a log-rank (Mantel-Cox) test.

To assess the *in vivo* efficacy of 4H1128ζ-IL12 T cells, we injected C57BL/6 mice with 1 × 10^7^ ID8-Muc16^ecto^ cells intraperitoneally (i.p.) followed by treatment with 2 × 10^6^ CAR T cells i.p. 35 days (D35) after tumor inoculation (Fig. 2a). Survival was significantly enhanced in 4H1128ζ-IL12 treated mice compared to animals treated with 4H1128ζ (median OS: Not reached vs 52 days. *p = 0.02.), 1928ζ (median OS: 50 days) or 1928ζ-IL12 (median OS: 55 days). IL-12 could potentially mediate autocrine effects via IL-12 receptor (IL-12R) signaling on 4H1128ζ-IL12 T cells or via paracrine mechanisms on other tumor associated cells such as macrophages. To explore the effects of autocrine signaling in mediating the *in vivo* efficacy of 4H1128ζ-IL12 T cells, donor T cells used for retroviral transduction were derived from IL-12R knock out (IL-12R^−/−^) splenocytes. CAR T cells derived from IL-12R^−/−^ splenocytes are unable to respond to IL-12. In D35 tumor-bearing mice treated with IL-12R^−/−^ CAR T cells, the survival advantage conferred by 4H1128ζ-IL12 was lost (Fig. 2b), underscoring the significance of IL-12 autocrine activity on 4H1128ζ-IL12 T cells in promoting survival. Previous studies evaluating the efficacy of adoptively transferred T cells in syngeneic models of ovarian peritoneal carcinomatosis have reported treatment of tumor-bearing mice at day 7, 28 and 35. In our i.p peritoneal carcinomatosis model, the median survival of untreated and CD19- CAR T cell tumor-bearing mice is 50–60 days. We sought to determine if 4H1128ζ-IL12 T cells would remain efficacious at very advanced stages of disease. To test this hypothesis, we extend the treatment date of tumor-bearing mice from 35 to 42 days (D42) and even at this stage of disease, 4H1128ζ-IL12 T cells continued to impart a survival advantage compared to 4H1128ζ (Fig. 2c) (97 vs 50.5 days, *p < 0.01).

### 4H1128ζ-IL12 T cells expand, alter the ascitic cytokine tumor microenvironment and demonstrate increased cytotoxicity ***in vivo***

**Figure 3:**
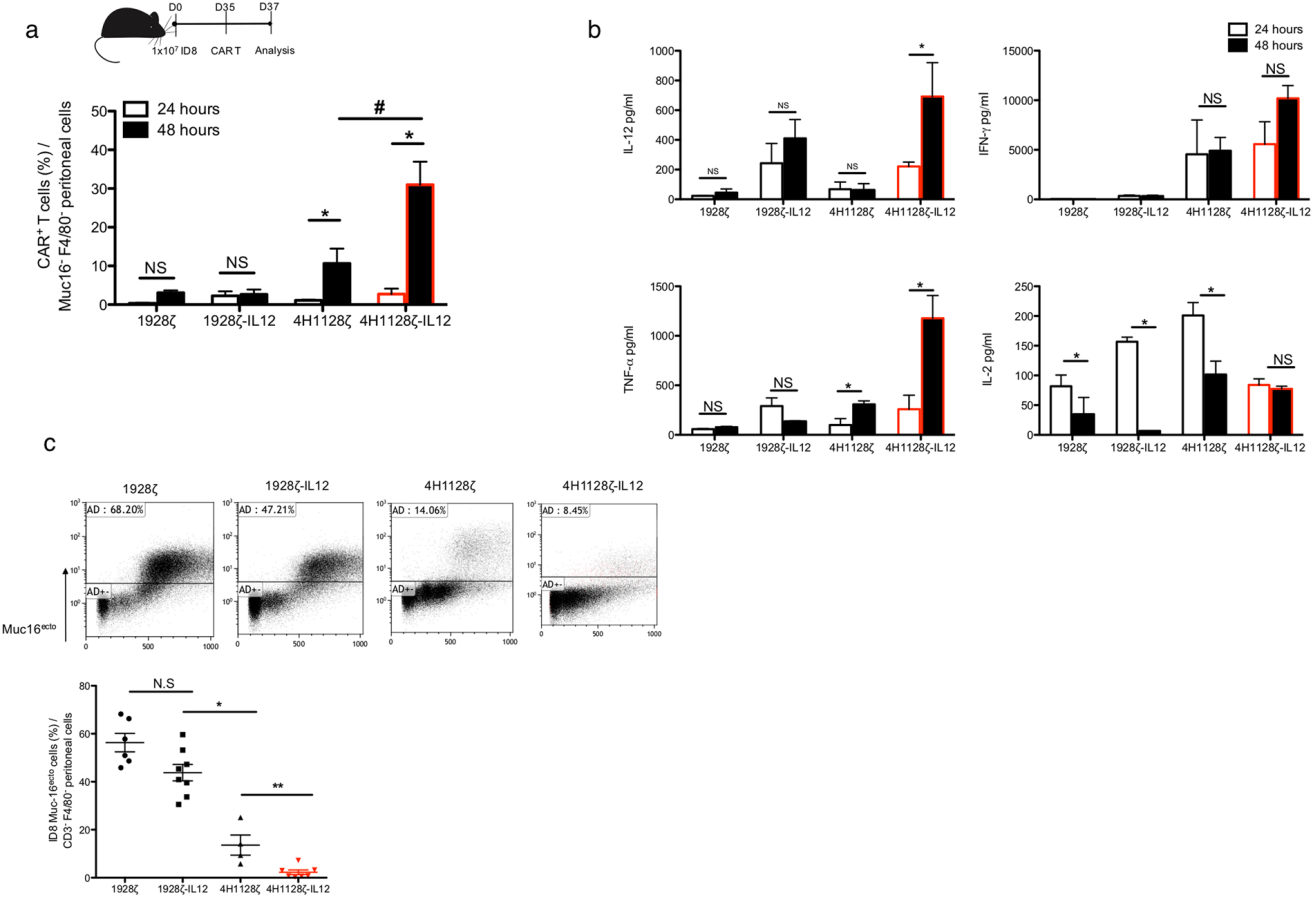
4H1128ζ-IL12 CAR T cells expand better, secrete inflammatory cytokines and eradicate tumor cells *in vivo*. **(a)** Female WT mice between 6–8 weeks old were inoculated with 1 × 10^7^ ID8-Muc16^ecto^ cells i.p and treated 35 days later with 2 × 10^6^ CAR T cells i.p. Mice were subsequently euthanized 24 hr and 48 hr after CAR T cell injection and peritoneal washes were performed. FACS analysis was performed on recovered peritoneal cells and CAR+ T cells were gated on Muc16^−^ and F4/80^−^ cells. 4H1128ζ (*p = 0.01), 4H1128ζ-IL12 (*p = 0.04), ^#^p < 0.05. Data are plotted as mean ± SEM. Data shown are pooled results from 3 independent experiments. **(b)** Cytokine analysis performed on peritoneal fluid samples obtained from D35 tumor-bearing mice treated with CAR T cells as in (**a**). IL-12: (*p < 0.01), TNF-α (*p < 0.01), and IL-2: 1928ζ (*p = 0.031), 1928ζ-IL12 (*p < 0.01), 4H1128ζ (*p < 0.01). Data are plotted as mean ± SEM. Results are pooled from 3 independent experiments. **(c)** WT female mice between 6–8 weeks old inoculated with 1 × 10^7^ ID8-Muc16^ecto^ cells i.p were treated 35 days later with 2 × 10^6^ CAR T cells i.p and subjected to peritoneal fluid analysis 48 hr after T cell infusion for the number of viable ID8-Muc16^ecto^ cells. *p < 0.05, **p < 0.001. Data are plotted as mean ± SEM. Results are pooled from 3 independent experiments. Statistics performed using unpaired two-sided T test.

*In vivo* expansion, cytokine production and cytotoxicity are required and necessary for effective CAR T cell therapy. We investigated the potential of these factors as the mechanisms by which 4H1128ζ-IL12 T cells improve overall survival. Tumor-bearing mice that had been inoculated 35 days prior (D35) with ID8-Muc16^ecto^ i.p were treated with a single i.p infusion of 1928ζ, 1928ζ-IL12, 4H1128ζ or 4H1128ζ-IL12 T cells. Twenty-four and 48 hr after treatment, the peritoneal cavities of these animals were sampled (Fig. 3). The percentage of CAR T cells (CD3^+^ CAR^+^) recovered from peritoneal washes were quantified (Fig. 3a). At 24 hr, there is limited expansion of all CAR T cells but after 48 hr, there is significantly increased *in vivo* expansion of 4H1128ζ (*p = 0.01) and 4H1128ζ-IL12 (*p = 0.04) T cells (Fig. 3a). At 48 hr, 4H1128ζ-IL12 T cells show increased *in vivo* expansion compared to 4H1128ζ T cells (^#^p < 0.05). Cytokine analysis performed on peritoneal fluid samples obtained at 24 hr and 48 hr after CAR T cell infusion showed increased levels of IL-12 (*p < 0.01), and TNF-α (*p < 0.01) over time in mice treated with 4H1128ζ-IL12 T cells (Fig. 3b). We did not see any significant increases in i.p. IL-2 levels in mice treated with 4H1128ζ-IL12 T cells despite robust *in vivo* expansion (Fig. 3a). In fact, IL-2 levels remained steady over this period suggestive of a steady state or equilibrated level of IL-2 production and utilization by 4H1128ζ-IL12 T cells. In contrast, IL-2 levels decreased in animals treated with 4H1128ζ, 1928ζ, and 1928ζ-IL12 T cells (*p < 0.01, p < 0.01, p = 0.031, respectively), possibly due to blunted proliferation and increased apoptosis by these cells in the ascitic microenvironment (Fig. 1g). Finally, we hypothesized that increased *in vivo* expansion and secretion of increased inflammatory cytokines in the ascitic microenvironment by 4H1128ζ-IL12 T cells would lead to reduction in the amount of viable tumor cells in the peritoneum. Indeed, we found that 48 hr after treatment of D35 ID8-Muc16^ecto^ tumor-bearing mice with 4H1128ζ-IL12 T cells, there were significantly decreased levels of tumor cells in peritoneal washes obtained from these animals compared to mice treated with 4H1128ζ T cells (*p < 0.001) (Fig. 3c).

### 4H1128ζ-IL12 T cells express decreased exhaustion markers and upregulate inflammatory genes in the ascitic microenvironment

**Figure 4:**
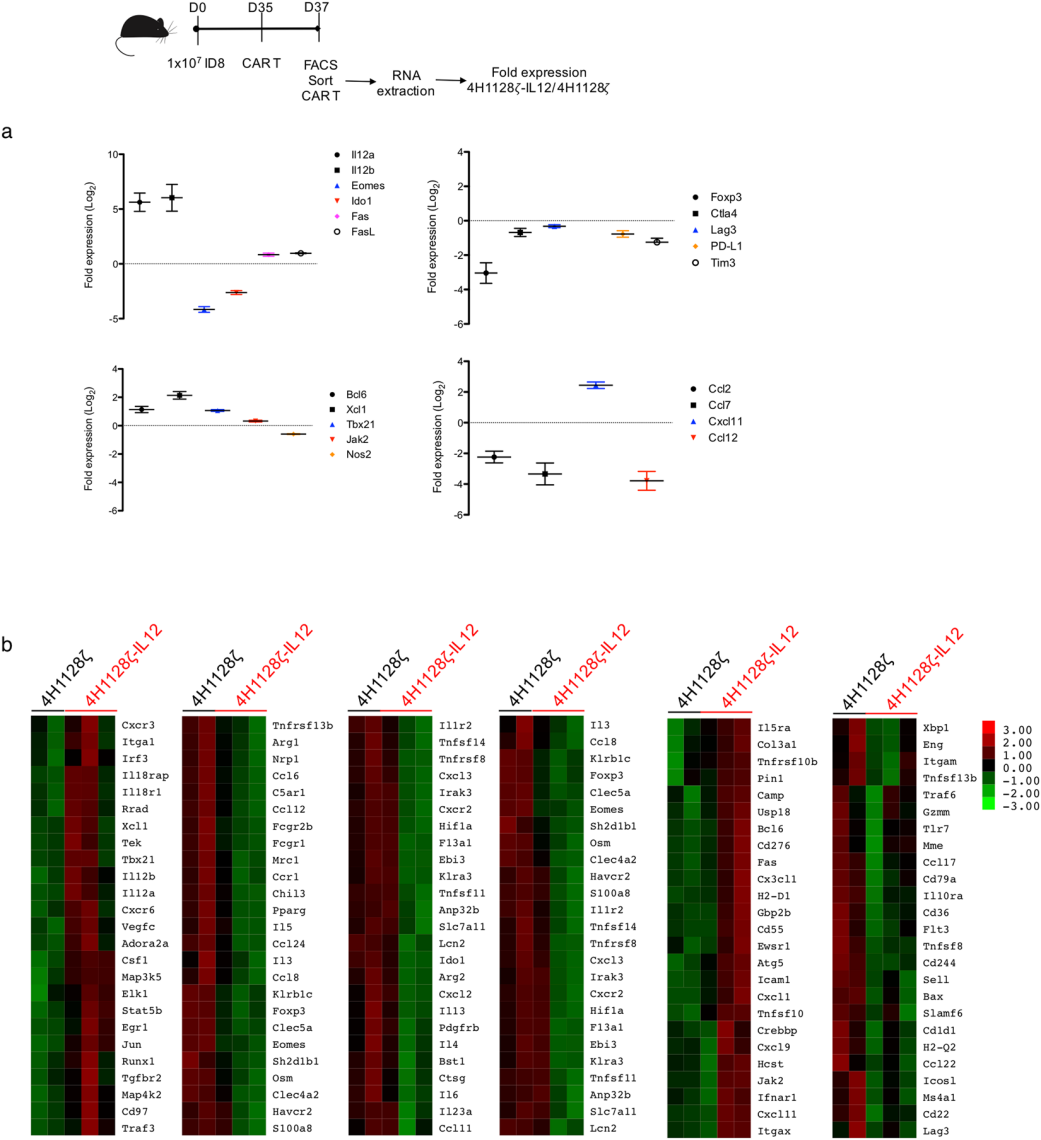
4H1128ζ-IL12 CAR T cells express decreased exhaustion markers. **(a)** Differential expression of statistically significant (p < 0.05) genes between pooled recovered CAR T cells from 4H1128ζ-IL12 and 4H1128ζ- treated mice. mRNA was extracted from pooled CAR T cells from 2 mice and performed in duplicate for 4H1128ζ and in triplicate for 4H1128ζ-IL12. n = 4 (4H1128ζ), n = 6 (4H1128ζ-IL12). Data presented as fold change of 4H1128ζ-IL12/4H1128ζ and plotted as mean ± SEM. **(b)** Heatmap representation of differentially expressed genes between recovered 4H1128ζ-IL12 and 4H1128ζ CAR T cells.

To further delineate the autocrine effects underlying 4H1128ζ-IL12 T cell efficacy, we treated D35 tumor-bearing mice with either 4H1128ζ or 4H1128ζ-IL12 T cells and retrieved the CAR T cells after 48 hr by peritoneal lavage. This time point was chosen based on both *in vitro* and *in vivo* data suggesting that the most dramatic difference between 4H1128ζ or 4H1128ζ-IL12 occurred around 48 hr. These cells were immediately flow sorted for CD3^+^ CAR^+^ cells and RNA extracted from recovered 4H1128ζ and 4H1128ζ-IL12 T cells were subjected to a 770-gene nanoString PanCancer Immune Profiling panel for transcriptome analysis. We identified genes that demonstrated statistical significance between 4H1128ζ and 4H1128ζ-IL12 T cells (p < 0.05) and plotted these as log_2_ fold change of 4H1128ζ-IL12 over 4H1128ζ. As shown in Fig. 4 and Supplementary Fig. 1, several genes associated with inflammation, exhaustion and chemotaxis were significantly different at 48 hr between armored and second generation CAR T cells. Compared to 4H1128ζ, 4H1128ζ-IL12 T cells showed significantly increased expression of IL-12 (IL-12a, IL-12b) as would be expected. There was also decreased expression of Eomes, FoxP3, Ctla4, Lag3, Tim3 and PD-L1 in 4H1128ζ-IL12 T cells compared to 4H1128ζ, all of which are consistent with decreased exhaustion. We also found significant upregulation of T-box transcription factor (Tbx21), a T-box domain gene upstream of inflammatory Th1 cytokines such as IFN-γ. 4H1128ζ-IL12 T cells also showed elevated levels of Fas and FasL, molecules that have been shown to be mediate cytotoxic T cell mediated killing via apoptosis. Chemokine family members associated with monocyte recruitment such as CCl2, CCl7 and CCl12 were downregulated in 4H1128ζ-IL12 compared to 4H1128ζ T cells. Genes involved in various aspects of T cell metabolism such as Indoleamine-pyrrole 2,3-dioxygenase (IDO) and Nitric oxide synthase (Nos2) were relatively downregulated in 4H1128ζ-IL12 T cells with implications for resisting tryptophan deprivation and activation induced cell death respectively^44, 45^.

### 4H1128ζ-IL12 T cells do not recruit endogenous T cells to the peritoneum or depend on endogenous cytotoxic cells

IL-12 has been shown to recruit cytotoxic T cells to sites of inflammation and cancer^46^. Based on these reports, we examined recruitment of endogenous T cells to the peritoneum of tumor-bearing mice treated with either 4H1128ζ or 4H1128ζ-IL12 T cells. To do this, we inoculated D35 ID8-Muc16^ecto^ -bearing C57BLK/6 Ly5.1 mice with 4H1128ζ or 4H1128ζ-IL12 T cells derived from WT C57BL/6 Thy1.2 splenocytes. Peritoneal washes were performed 48 hr after CAR T cell infusion and stained for CD3^+^ Ly5.1^+^ cells. As shown in Supplementary Fig. 2, less endogenous T cells were recruited to the peritoneum of mice treated with 4H1128ζ-IL12 T cells compared to 4H1128ζ after 48 hr (1.7% vs 3.5%, *p = 0.04). Host-derived effector T cells could potentially exert their effects before or after the 48 hr window we examined, hence, we used survival in CD8 knockout (CD8^−/−^) and IFN-γ knockout (IFN-γ^−/−^) mice as surrogates for host-derived effector contribution. Treatment of D35 tumor-bearing CD8^−/−^ mice with 4H1128ζ-IL12 T cells did not compromise its efficacy compared to 4H1128ζ (median OS; not reached vs 52 days, ^#^p = 0.001) or untreated mice (Supplementary Fig. 2). Similarly, absence of host IFN-γ did not impede 4H1128ζ-IL12 function compared to 4H1128ζ (median OS; not reached vs 56 days, *p < 0.001). There was no significant difference in survival between CD8^−/−^ and IFN-γ^−/−^ mice treated with 4H1128ζ-IL12 (N.S) (Supplementary Fig. 2).

### 4H1128ζ-IL12 T cells decrease tumor associated macrophages in a Fas/FasL dependent manner

**Figure 5:**
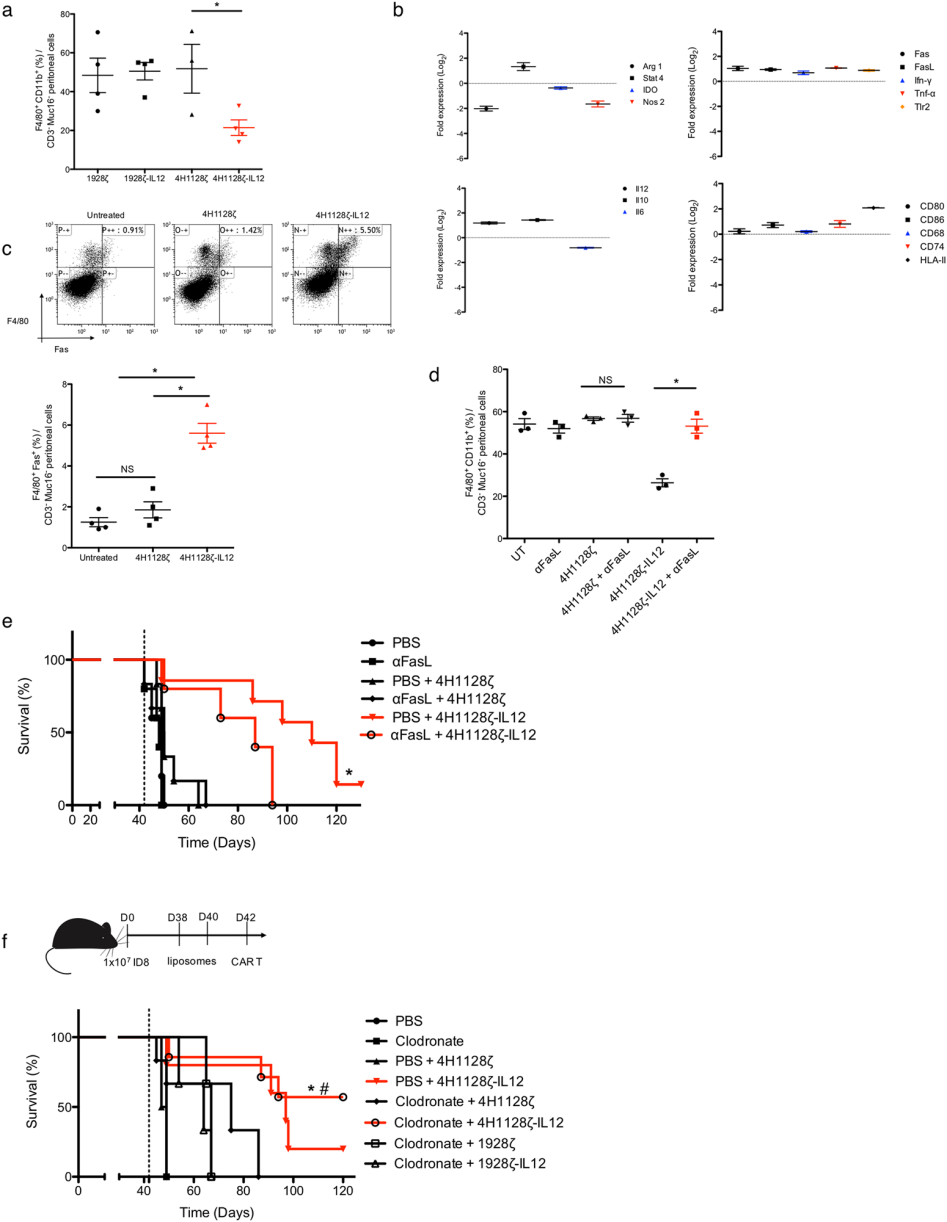
4H1128ζ-IL12 CAR T cells phenotypically alter and delete tumor associated macrophages. (**a)** Peritoneal washes from tumor-bearing mice treated with 4H1128ζ-IL12 compared to 4H1128ζ CAR T cells and assessed for F4/80^+^ CD11b^+^ TAM (*p < 0.05). Data are plotted as mean ± SEM. Data shown are pooled results from 3 independent experiments. **(b)** Statistically significant (p < 0.05) differential gene expression between F4/80^+^ CD11b^+^ TAM recovered from mice treated with 4H1128ζ-IL12 vs 4H1128ζ. mRNA was extracted from pooled TAM from 2 mice per group after FACS sorting. n = 6 (4H1128ζ), n = 6 (4H1128ζ-IL12). Data presented as fold change of 4H1128ζ-IL12 TAM / 4H1128ζ TAM and plotted as mean ± SEM. **(c)** Analysis of Fas expression on F4/80^+^ TAM recovered from animals treated with CAR T cells (*p < 0.01). Data are plotted as mean ± SEM. Data shown are pooled results from 3 independent experiments. **(d)** TAM recovery from day 35 tumor-bearing mice treated with anti-FasL (αFasL) neutralizing antibody 1 day prior to CAR T infusion, *p < 0.01. Data are plotted as mean ± SEM. Data shown are pooled results from 3 independent experiments. **(e)** Female WT mice age 6–8 weeks inoculated with 1 × 10^7^ ID8-Muc16^ecto^ cells i.p followed by treatment with 250 μg αFasL i.p on day 41 and CAR T cells on day 42. *p = 0.04. Number of mice per group: 5 (PBS), 5 (αFasL), 6 (PBS + 4H1128ζ), 6 (αFasL + 4H1128ζ), 7 (PBS + 4H1128ζ-IL12), 6 (αFasL + 4H1128ζ-IL12). Results are pooled from 2 independent experiments. **(f)** WT tumor-bearing female mice between 6–8 weeks old were treated with 2 doses of clodronate (Clod) or PBS liposomes on Day 38 and 40. Subsequently, mice were treated with CAR T cells on D42. *p < 0.05. Number of mice per group: 5 (PBS), 5 (Clod), 5 (PBS + 4H1128ζ), 5 (PBS + 4H1128ζ-IL12), 6 (Clod + 4H1128ζ), 7 (Clod + 4H1128ζ-IL12), 5 (Clod + 1928ζ), 5 (Clod + 1928ζ-IL12). Results are pooled from 3 independent experiments. Statistical analysis performed using a log-rank (Mantel-Cox) test.

Tumor associated macrophages (TAMs) represent a significant proportion of the ascites tumor microenvironment in ovarian cancer. These cells exert suppressive effects on T cells via multiple mechanisms and have been associated with poor outcomes^47^. Based on prior data showing the effects of IL-12 on tumor associated macrophages^48^, we evaluated the hypothesis that IL-12 secreted by 4H1128ζ-IL12 T cells was potent enough to alter the cellular microenvironment as early as 48 hr. To this end, we treated D35 tumor-bearing mice with 4H1128ζ-IL12, 4H1128ζ and CD19-directed T cells and evaluated the number of recovered TAMs after 48 hr. We found significantly decreased peritoneal F4/80^+^ CD11b^+^ TAMs in 4H1128ζ-IL12 treated mice after 48 hr compared to the other conditions (Fig. 5a). To further investigate genotypic changes induced by IL-12, F4/80^+^ CD11b^+^ TAMs were harvested and flow sorted from peritoneal washes of 4H1128ζ-IL12 and 4H1128ζ treated mice. Sorted cells from each experimental group was subjected to RNA extraction and transcriptome analysis. Genes that demonstrated statistical significance between 4H1128ζ and 4H1128ζ-IL12 T cells (p < 0.05) were identified and plotted as log_2_ fold change of 4H1128ζ-IL12 over 4H1128ζ. IL-12 secreted from 4H1128ζ-IL12 T cells lead to downregulation of Arg1, decreased levels of which are associated with a pro-inflammatory M1 TAM phenotype (Fig. 5b). Levels of IL-12, IFN-γ, TNF-α and Toll-like receptor 2 (Tlr2) were significantly upregulated. Stat4, a mediator of IL-12 function, was also differentially upregulated in 4H1128ζ-IL12 treated mice. Additional transcriptome changes are shown in Supplementary Fig. 1. We validated upregulation MHC-II in TAMs recovered from mice treated with 4H1128ζ-IL12 T cells (Supplementary Fig. 1) in addition to decreased intracellular and secreted IL-6 and IL-10 (Supplementary Fig. 1). We also found upregulated Fas on TAMs recovered from 4H1128ζ-IL12 treated mice. Fas/FasL engagement has been reported as a mechanism via which T cells eliminate macrophages as means of controlling inflammation^49^. We verified increased cell surface expression of Fas on TAMs recovered 48 hr after 4H1128ζ-IL12 treatment (Fig. 5c). Based on gene expression profile data that showed upregulation of FasL on 4H1128ζ-IL12 T cells (Fig. 4), we hypothesized that decreased TAMs were a result of Fas engagement on TAMs by FasL on 4H1128ζ-IL12 T cells. To test this hypothesis directly, we evaluated the presence of TAMs after a single dose of an anti-FasL blocking antibody (αFasL) administered 24 hr prior to CAR T administration. We found that blocking the Fas/FasL pathway did not lead to any significant differences in 4H1128ζ T cell-treated mice after 48 hours but led to recovery of TAM levels in 4H1128ζ-IL12 treated mice, effectively rescuing the phenotype (Fig. 5d). Furthermore, pre-treatment with αFasL led to decreased survival in D42 tumor-bearing mice treated with 4H1128ζ-IL12 (Fig. 5e). To evaluate if TAM depletion contributed to the survival advantage seen in mice treated with 4H1128ζ-IL12, we pretreated D42 tumor-bearing mice with 2 i.p. injections of either clodronate or pbs-liposomes preceding CAR T cell administration. Clodronate depletion led to improvements in survival in both 4H1128ζ and 4H1128ζ-IL12 treated mice, but this improvement was only statistically significant in the 4H1128ζ-IL12 treatment group (p < 0.05). These results show that decreased levels of F4/80^+^ CD11b^+^ TAMs improve outcome and suggest that Fas/FasL mediated depletion of TAMs by 4H1128ζ-IL12 T cells could potentially contribute to survival.

### 4H1128ζ-IL12 T cells resist endogenous PD-L1-mediated inhibition by tumor cells

**Figure 6:**
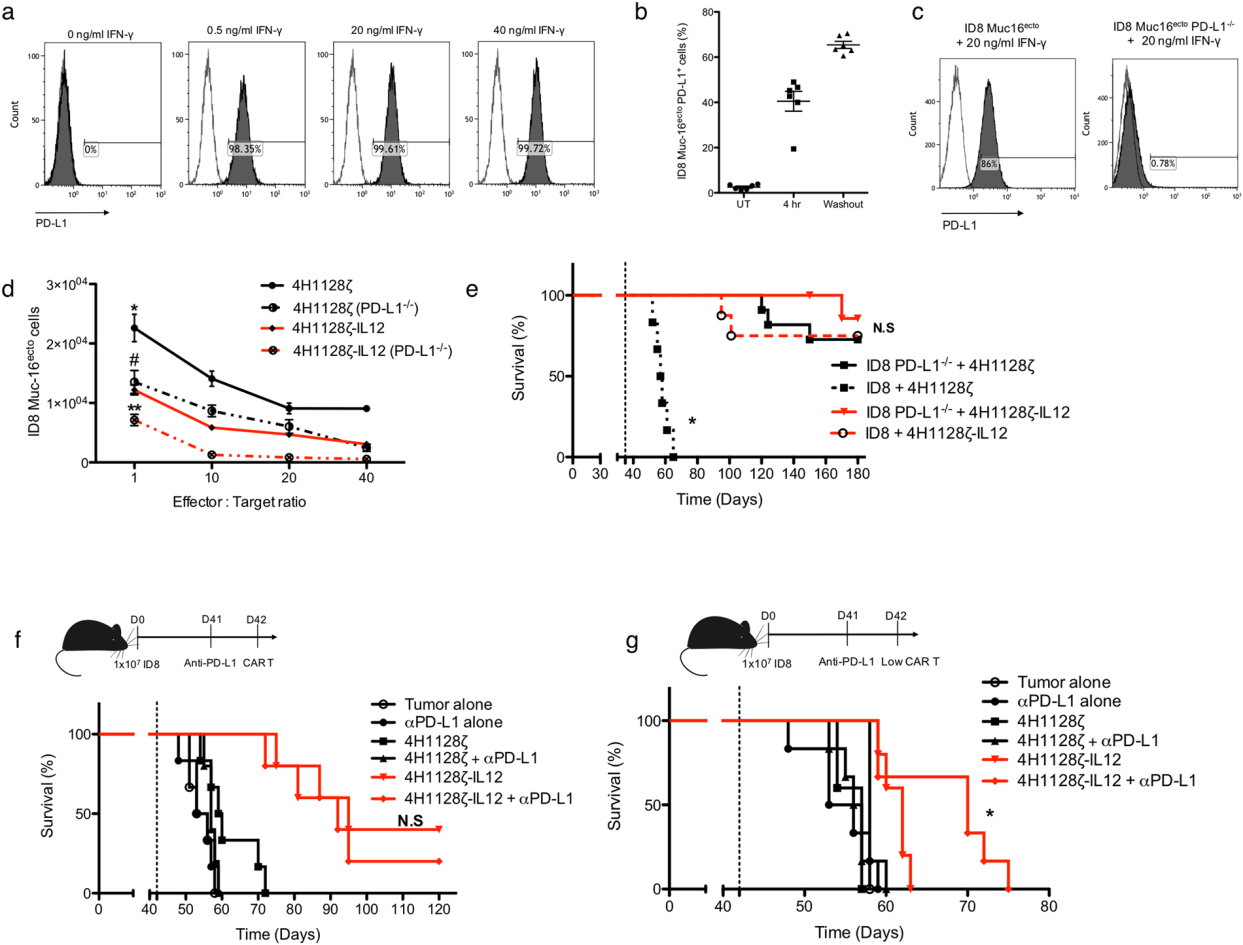
4H1128ζ-IL12 CAR T cells overcome endogenous tumor PD-L1 suppression. (**a**) ID8-Muc16^ecto^ cells cocultured with various concentrations of recombinant IFN-γ and incubated for 16 hr. **(b)** ID8-Muc16^ecto^ cells incubated with recombinant IFN-γ and assessed for PD-L1 upregulation after 4 hr of exposure. Similarly, tumor cells were exposed to IFN-γ for 16 hr, washed and then incubated in media without IFN-γ for 24 hr. Data are plotted as mean ± SEM. Data shown are pooled from 2 independent experiments. **(c)** Murine PD-L1 gene was genetically deleted using CRISPR-Cas9 and ID8-Muc16^ecto^ PD-L1^−/−^ cells were screened via stimulation with IFN-γ for 16 hr. Data shown are pooled from 2 independent experiments. **(d)** ID8-Muc16^ecto^ PD-L1^−/−^ and ID8-Muc16^ecto^ cells cocultured with CAR T cells at the indicated E:T ratios for 16 hr. The number of residual tumor cells from each condition is shown, *p < 0.05, **p < 0.05, ^#^N.S. Data shown are pooled from 2 independent experiments. **(e)** D42 tumor-bearing mice inoculated with ID8-Muc16^ecto^ or ID8-Muc16^ecto^ PD-L1^−/−^ cells and treated with indicated CAR T cells. *p < 0.001. n = 8 mice per group. Results pooled from 2 independent experiments. **(f)** D42 tumor-bearing mice previously inoculated with ID8-Muc16^ecto^ cells were pretreated on D41 with 250 μg of anti-PD-L1 blocking antibody (αPD-L1) prior to i.p infusion of 2 × 10^6^ 4H1128ζ-IL12 or 4H1128ζ CAR T cells. Number of mice per group: 3 (Tumor), 6 (αPD-L1), 6 (4H1128ζ), 6 (4H1128ζ + αPD-L1), 6 (4H1128ζ-IL12), 6 (4H1128ζ-IL12 + αPD-L1). **(g)** D42 tumor-bearing mice pretreated with 250 μg αPD-L1 followed by 2 × 10^5^ CAR T cells, *p < 0.05. Number of mice per group: 3 (Tumor), 6 (αPD-L1), 5 (4H1128ζ), 6 (4H1128ζ + αPD-L1), 5 (4H1128ζ-IL12), 6 (4H1128ζ-IL12 + αPD-L1). Results are pooled from 2 independent experiments. Statistical analysis performed using a log-rank (Mantel-Cox) test.

Another mechanism via which tumor cells can evade the immune system is by upregulation of PD-L1. In particular, elevated PD-L1 has been reported in patients with ovarian cancer and its overexpression is correlated with poor outcomes^19^. PD-L1 expression has also been shown to be induced by IFN-γ, a major inflammatory cytokine which mediates some of the effects of activated cytotoxic T cells^50, 51^. We verified that our ID8-Muc16^ecto^ cells upregulated PD-L1 in response to IFN-γ (Fig. 6a). We also found induction of PD-L1 after as little as 4 hr of exposure to IFN-γ (Fig. 6b) with persistent expression after a 24 hr washout period of the cytokine (Fig. 6b). Additionally, we found that only ascites containing IFN-γ was capable of upregulating PD-L1 (Supplementary Fig. 3). To further evaluate the contribution of PD-L1 upregulation to the suppression of CAR cytotoxicity, we genetically silenced PD-L1 in our ID8-Muc16^ecto^ cells using CRISPR (ID8-Muc16^ecto^ PD-L1^−/−^) and validated that these cells were unresponsive to IFN-γ exposure (Fig. 6c). We then performed cytotoxicity assays comparing the efficacy of 4H1128ζ and 4H1128ζ-IL12 T cells against ID8-Muc16^ecto^ and ID8-Muc16^ecto^ PD-L1^−/−^ cells (Fig. 6d). Deletion of PD-L1 improved efficacy of both 4H1128ζ and 4H1128ζ-IL12 over WT ID8-Muc16^ecto^. Strikingly, PD-L1 deletion improved cytotoxicity of 4H1128ζ T cells to the same level as the cytotoxicity of 4H1128ζ-IL12 against unmodified ID8-Muc16^ecto^ (Fig. 6d). *In vivo*, treatment of D35 ID8-Muc16^ecto^ PD-L1^−/−^ tumor-bearing mice with 4H1128ζ T cells rescued the attenuated efficacy seen with mice inoculated with WT ID8-Muc16^ecto^ at the same time point (*p < 0.001, solid black line vs. dotted black line) (Fig. 6e). D35 ID8-Muc16^ecto^ PD-L1^−/−^ tumor-bearing mice treated with 4H1128ζ-IL12 T cells did not exhibit significant differences in median overall survival compared to WT ID8-Muc16^ecto^ treated with 4H1128ζ-IL12 T cells (N.S, solid red line vs. dotted red line). Next, we sought to determine if administration of a single dose of αPD-L1 immune checkpoint inhibition could further enhance survival in WT ID8-Muc16^ecto^ tumor-bearing mice treated with 4H1128ζ-IL12 T cells. We pretreated D42 tumor-bearing mice with a single dose of blocking αPD-L1 antibodies i.p 1 day (D41) prior to the administration of CAR T cells, followed by CAR T infusion. A single infusion of the antibody alone did not yield any benefits on survival compared to untreated mice (Fig. 6f). Antibody therapy marginally increased survival in animals treated with full dose (2 × 10^6^ CAR^+^ T cells) 4H1128ζ T cells and 4H1128ζ-IL12 T cells although these differences were not statistically significant. However, when αPD-L1-pretreated animals were treated with 1 log_10_ fold reduction of CAR T cells (2 × 10^5^ CAR^+^ T cells), 4H1128ζ-IL12 T cell efficacy was significantly improved (*p < 0.05) (Fig. 6g). Taken together, these results suggest that despite inducible PD-L1 upregulation on tumor cells, 4H1128ζ-IL12 T cells retain efficacy.

### 4H1128ζ-IL12 T cells do not show any measurable toxicity in syngeneic C57BL/6 mice

Finally, we sought to evaluate whether administration of 4H1128ζ-IL12 T cells would be toxic to mice in a syngeneic C57BL/6 model. Despite several limitations to the assessment of toxicity in mice and the correlation of those toxicities to humans, acute IL-12 toxicity has been reported in mice^34^ and humans^31^. Therefore, we undertook pharmacotoxicity studies in tumor (T) and non-tumor (NT) bearing mice treated with 4H1128ζ-IL12 T cells. Since we were unable to detect any significant systemic IL-12 levels in tumor-bearing mice treated with i.p 4H1128ζ-IL12 T cells 7 days after CAR T cell administration (Supplementary Fig. 4), we treated T and NT mice with 4H1128ζ-IL12 T cells administered both i.v and i.p and evaluated body weights, blood chemistries and various hematologic parameters. As shown in Supplementary Fig. 4, there were no significant differences in the weights of T and NT mice treated with i.v/i.p 4H1128ζ-IL12 T cells over 30 days after treatment. We did not detect any significant laboratory abnormalities 3 days and up to 16 days after 4H1128ζ-IL12 treatment (Supplementary Fig. 4). Although we saw a transient statistically significant decrease in WBC and lymphocyte counts in tumor-bearing mice treated with 4H1128ζ-IL12 after 3 days, these values were still within reference ranges (WBC: 1.8–10.7 K/μL; lymphocytes: 0.9–9.3 K/μL) and spontaneously resolved by day 16.

## Discussion

Recombinant IL-12 has been explored as a therapeutic agent for multiple malignancies including melanoma^30^, renal cell cancer^31^ and ovarian cancer^32, 33^. In ovarian cancer, i.v IL-12 administration on a phase II clinical trial led to a partial response in 1 patient (3.8%), and stable disease in 13 patients (50%)^32^. Antitumor efficacy of i.p IL-12 has also been reported^33, 52^. In this phase II report, i.p IL-12 administration led to increased peritoneal IFN-γ, TNF-α, and peritoneal tumor apoptosis. However, myelosuppression^32^, poor tolerability^33^, hemodynamic instability^35^ and treatment-related fatalities^31^ have dampened enthusiasm for systemic recombinant IL-12 administration. Many of these toxicities are felt to be dose and schedule related^31^. In this report, we present data on safety and mechanisms of efficacy of CAR T cells genetically modified to secrete IL-12 in an aggressive disseminated mouse model of ovarian cancer.

These armored CAR T cells show increased perforin and granzyme B levels associated with increased cytotoxic activity *in vitro*. Furthermore, autocrine IL-12 function led to increased *in vivo* expansion, *in vitro* proliferation and cytotoxicity in ascites. At 48 hr, IL-12 armored CAR T cells exhibited transcription markers suggestive of a trend towards decreased exhaustion compared to second generation CAR T cells after i.p transfer. The immunosuppressive capacity of ascites has been well described^41–43^, and our data clearly demonstrates the negative effects of this ascitic microenvironment on adoptively transferred second generation CAR T cells. Beyond the autocrine augmentation provided by IL-12, we also found increased levels of peritoneal IFN-γ, TNF-α and superior eradication of peritoneal tumor cells similar to clinical reports of recombinant IL-12 administration^33, 52^. This suggests that armored CAR T cells produce biologically active levels of IL-12 to remodel the peritoneal cytokine microenvironment and improve CAR T cell efficacy.

Another phenotypic readout of cytokine remodeling of the peritoneal microenvironment is evident from the alterations of TAMs recovered from mice treated with IL-12 armored CAR T cells. The potential for IL-12 delivered to the tumor microenvironment to enhance T cell efficacy has been shown in a mouse melanoma/PMEL model^27^. Reprogramming of TAMs in response to IL-12 has also been reported^27^ and potentiation of macrophage-derived IL-12 and IFN-γ in response to IL-12 has been described^53^. In this study, we report a pro-inflammatory TAM phenotype characterized by downregulated Arg1 and upregulated IFN-γ, TNF-α, Tlr2 and MHC-II. Additionally, we show that armored CAR T cells also facilitate eradication of TAMs via Fas/FasL. To reconcile these findings with our described phenotypic M1-like alterations, we propose that IL-12 diffusing throughout the peritoneum leads to the phenotypic skewing of TAMs, however, TAMs that encounter armored CAR T cells are induced to undergo apoptosis as previously reported^49^. The nature of this interaction is likely temporally and spatially regulated and beyond the scope of this report. What is clear is that reduction of TAMs is beneficial as shown by our depletion studies.

Lymphocyte-derived IFN-γ has been shown to upregulate PD-L1 on tumors^50^, leading to arrest of infiltrating and adoptively transferred T cell function. We show that this process occurs relatively early and is durable even after removal of IFN-γ. As predicted, second generation CAR T cells are not as effective at eradicating PD-L1^+^ tumors *in vitro* and *in vivo* compared to armored CAR T cells. Furthermore, CRISPR-mediated deletion of PD-L1 rescued the *in vitro* cytotoxicity defect of second generation CAR T cells to the level of unmodified tumor cells treated with armored CAR T cells. *In vivo*, ablation of PD-L1 expression on tumor cells dramatically improved efficacy in second generation CAR-treated animals without conferring any significant additional advantage to armored CAR T cells. These findings are significant, as they suggest that second generation CAR T cell approaches for disseminated ovarian cancer might not be as efficacious as expected in clinical trials. Surprisingly, pretreatment with an anti-PD-L1 checkpoint inhibitor did not improve armored CAR T cell efficacy. Only when the cell therapy dose was reduced by an order of magnitude could a difference be elicited. Indeed, it would appear that armored CAR T cells are potentially effective against PD-L1^+^ tumor cells at this low dose and pretreatment with checkpoint inhibition only serves to block the non-tumor PD-L1^+^ cells such as macrophages and stromal cells that are not directly targeted by the CAR T cell but never-the-less suppress CAR T cell activity. One potential application of this finding is the use of checkpoint inhibition as a part of a preconditioning regimen prior to armored CAR administration. Alternatively, preconditioning with checkpoint blockade might allow for lowering of the dose of administered CAR T cells without significantly compromising efficacy and improving safety.

Mice are imperfect surrogates for assessing toxicity of adoptive immunotherapy. However, IL-12 toxicity in mice^34^ has been described and correlated with toxicity in clinical trials^35^. We were unable to detect detrimental levels of IL-12 or IL-6 in tumor-bearing animals treated with armored CAR T cells. Additionally, we did not observe any significant weight loss in tumor-bearing mice treated with armored CAR T cells over a 30-day period. Detailed evaluation of murine blood chemistries, renal function, hepatic function and hematologic parameters did not reveal any signs of IL-12 toxicity. Although tumor-bearing mice treated with armored CARs had a short-lived decline in white blood cell count secondary to mild lymphopenia, these values were within reference values, recovered without intervention and did not negatively affect involved animals. To the best of our characterization, therapy with our IL-12 armored CARs does not incur toxicity in mice.

Clinical trials in ovarian cancer using folate receptor- directed CAR T cells did not lead to any clinical responses, likely secondary to an immunosuppressive tumor microenvironment^5^. Herein we report the efficacy of IL-12 secreting CAR T cells via enhanced proliferation, preserved cytotoxicity and protection from apoptosis in the presence of ascites. These benefits extend to improved expansion and cytotoxicity *in vivo*, leading to survival benefits in an advanced tumor-bearing mouse model. Furthermore, these cells reshape peritoneal cytokines and tumor associated macrophages towards a more inflammatory phenotype, and despite upregulation of PD-L1, armored CAR T cells retain antitumor efficacy. Lastly, extensive murine toxicology studies do not show any adverse effects of armored CAR T cell therapy. Based on the summation of these findings and evidence of efficacy against human ovarian tumors in SCID-beige models^37^, we are currently undertaking a phase I clinical trial to evaluate safety of armored IL-12 secreting CAR T cells in Muc-16^+^ ovarian carcinoma (NCT02498912).

## Materials and Methods

### Generation of retroviral constructs

SFG-1928ζ and 1928ζ-IL12 vectors were derived from previously described SFG-19ζ constructs^38^. The 4H1128ζ-IL12 construct was derived from the published 4H1128ζ vector^36^. All constructs were verified by sequencing.

### Cell lines

Murine ovarian cancer lines ID8-Muc16^ecto^, and ID8-Muc16^ecto^ GFP-Luc were maintained in DMEM (Invitrogen, Grand Island, NY, USA) supplemented with 10% heat-inactivated fetal calf serum (FBS), 100 U/ml penicillin and streptomycin (P/S), and 2mM L-glutamine. ID8-Muc16^ecto^ Cas9 was maintained in DMEM supplemented with 10% FBS, P/S and 5 μg/ml blasticidin. ID8-Muc16^ecto^ PD-L1^−/−^ and ID8-Muc16^ecto^ PD-L1^−/−^ GFP-Luc were maintained in DMEM supplemented with 10% FBS, P/S, 5 μg/ml Blasticidin and 2 μg/ml puromycin. ID8-Muc16^ecto^ cells were validated using karyotype analysis and routinely checked for mycoplasma contamination. Phoenix ecotropic packaging cells (ATCC) producing 1928ζ, 1928ζ-IL12, 4H1128ζ, and 4H1129ζ-IL12 were maintained in DMEM with 10% FBS, L-glutamine, and penicillin/streptomycin^38^. PD-L1 CRISPR was performed with assistance from the Memorial Sloan Kettering Cancer Center RNAi core. The CRISPR-Cas9 guide-sequence to mouse PD-L1 (gene ID: 60533) was performed as per Doench *et al*.^53^. The forward (CACCGCTCAGCACAGCAACTTCAGG) and reverse (AAACCCTGAAGTTGCTGTGCTGAGC) sequences were packaged in a lentiviral vector with puromycin selection antibiotic. The CAS9 gene was packaged in a lentiviral backbone vector encoding blasticidin resistance. After antibiotic selection, PD-L1 knock down efficiency was assed via addition of recombinant IFN-γ and assessment for PD-L1 upregulation by FACS.

### T-cell isolation and gene transfer

Mouse T cells were isolated from splenocytes derived from C57BL/6, or IL-12R^−/−^ mice after euthanization as per our IUCAC protocol. Spleens were mechanically dissociated and incubated with ACK lysis buffer (Lonza) for 5 mins to lyse RBCs. The lysis reaction was quenched with RPMI 1640 medium (Gibco) supplemented with 10% heat-inactivated FBS (Atlanta Biologicals), nonessential amino acids, sodium pyruvate, HEPES (N-2-hydroxyethylpiperazine-N’-2-ethanesulfonic acid), L-glutamine, penicillin/streptomycin and 2-mercaptoethanol (RPMI-FBS10-ATOS) (Invitrogen). Resuspended cells were passed through a nylon wool fiber column (Polysciences, Warrington, PA) to isolate T cells. Purified mouse T cells were activated with CD3/CD28 beads, according to manufacturer’s instructions (Gibco, Thermo Fisher, Whaltman, MA) and cultured in RPMI-FBS10-ATOS plus 100 IU/ml recombinant human IL-2 (Proleukin, Novartis, Basel, Switzerland). The following day, T cells were spinoculated with retroviral supernatant on retronectin (Takara, Otsu, Shiga, Japan) coated plates at 3200 rpm at 30 °C for 60 min for 3 days. CAR expression was detected using Armenian hamster 12D11 antibody or an Alexa-Fluor647 conjugated hamster antibody that specifically binds the 4H1128z CAR (Monoclonal Antibody Facility, Memorial Sloan Kettering Cancer Center).

### Proliferation/Apoptosis/cytotoxicity assay

To assess proliferation, 1 × 10^6^ CAR + T cells were cocultured with a confluent plate of ID8-Muc16^ecto^ tumor cells. CAR T cell expansion was measured on days 1, 2, 5 and 7 by flow cytometry and counting beads (Ebioscience) according to manufacturer’s instructions. To determine apoptosis, CAR + T cells were cocultured with ID8-Muc16^ecto^ tumor cells at 1:1 effector to target ratio for 48 hr. Cells were stained with anti-CD3 (eBioscience) and anti-CAR antibodies and then labelled with annexin V (eBioscience) and DAPI. The percentage of apoptotic cells were determined as annexin V + population gated on CD3+ CAR+ cells. To measure cytotoxicity, CAR+ T cells were cocultured with ID8-Muc16^ecto^ GFP-Luc or ID8-Muc16^ecto^ PD-L1^−/−^ GFP-Luc at the indicated effector: target ratios for 16 hr and subsequently mixed with luciferase assay reagent (Promega). Luminescence of the lysates was analyzed using a plate spectrophotometer. These experiments were performed in the presence of either DMEM + 10% FBS or cell-free pooled ascites. Arginase activity assay on recovered peritoneal TAMs was performed according to manufacturer’s instruction (abcam: ab180877).

### Peritoneal cell recovery

For cell-free ascites collection, tumor-bearing mice between D35-D50 after tumor inoculation were euthanized as per protocol. The animals were sprayed with 70% ethanol and a 18G11/2 needle attached to a 10 ml syringe was used to aspirate the peritoneal fluid. Ascitic fluid was centrifuged at 2200 rpm × 15 mins at 4 °C and the supernatant was filtered through a 45-micron filter (Corning). Pooled ascitic fluid were either used for cytokine analysis or frozen at −80 °C for further use. For peritoneal cell characterization, the abdominal cavity of each treated tumor-bearing animal was lavaged with 7 ml of ice cold PBS. The retrieved fluid was centrifuged at 2200 rpm × 15 mins at 4 °C and the supernatant was either used for cytokine analysis or discarded. The pellet was resuspended in ACK lysis buffer (Lonza) for 5–10 mins and neutralized with FACS buffer (PBS + 2.5% FBS). The cells were washed 3 times with FACS buffer, counted and subjected to FACS analysis. For quantification of T cells, CD3+ CAR+ cells were represented as a fraction of Muc16− F4/80− peritoneal cells. Quantification of tumor cells are represented as the proportion of Muc16+ cells gated against CD3− F4/80− cells. Tumor associated macrophages are represented as a fraction of CD3− Muc16− peritoneal cells.

### Cytokine analyses

Collection of *in vivo* peritoneal cytokines described as above. Serum cytokines were measured from blood collected via retro-orbital bleeds from indicated animals and centrifuged to separate the serum fraction. For *in vitro* cytokine secretion experiments, CAR T cells were cocultured 1:1 with ID8-Muc16^ecto^ tumor cells (E:T ratio). Supernatant was collected after 16 hr of coculture and frozen at −80 °C until analysis. Cytokine detection was performed using the MILLIPLEX MAP Mouse Cytokine/Chemokine, Premixed 13 Plex kit, and the Luminex IS100 system.

### FACS analyses

Flow cytometric analyses were performed using Gallios Flow Cytometer with Kaluza software (Beckman Coulter, Brea, CA, USA). ID8-Muc16^ecto^ and CAR expression were detected using APC-conjugated anti-Muc16 and Armenian hamster 12D11 antibody respectively (Memorial Sloan Kettering Cancer Center monoclonal antibody facility). Mouse cells were stained with rat anti–mouse CD3 (PE/APC. eBioscience 145-2C11/17A2), F4/80 (APC. eBioscience MF48021), CD11b (PE. eBioscience 12-0112-82), PD-L1 (PE. eBioscience 12-5982-82), MHC-II (PE, Thermofisher M5/114.15.2), Granzyme B (PE, Thermofisher 12-8898-82), perforin (PE, Thermofisher 12-9392-82) FAS (PE. eBioscience 12-0951-81) and Ly5.1 (APC. Biolegend; A20). Tumor, mouse T cells or peritoneal cells were pelleted and washed 3 times with FACS buffer (PBS + 2.5% FBS). Cells were resuspended with the appropriate antibody, diluted in FACS buffer and incubated at 4 °C for 30 min in the dark. The cells were subsequently washed 3 times with cold FACS buffer and resuspended in 1X DAPI prior to FACS analysis. Flow sorting of CAR-positive T cells and TAMs was done using a BD Biosciences (San Jose, CA, USA) FACS ARIA IIu Special Order System with BD FACSDIVA software.

### Mice and ***in vivo*** model

Female C57BL/6, IFN-γ^−/−^ (B6.129S7-IFNγtm1Ts/J), CD8^−/−^ (B6.129S2-Cd8atm1Mak/J), Ly5.1 (B6.SJL-Ptprca Pepcb/BoyJ), and IL12R^−/−^ (B6.129S1-Il12rb2tm1Jm/J) mice age 6-8 weeks were purchased from Jackson Laboratories, Bar Harbor, ME. For day 35 (D35) and day 42 (D42) experiments, 1 × 10^7^ ID8-Muc16^ecto^ tumor cells were injected intraperitoneally (i.p.) on D0 and animals were treated with CAR T cells on D35 or D42 respectively. Mice treated with αFasL blocking antibody (R&D systems) were treated with 250 μg/mouse of antibody on D41 after tumor inoculation. Animals treated with PBS/Clodronate liposomes (Cedarlane, Amsterdam, The Netherlands) were injected with 200 μL/mouse of either liposome on D38 and D40 after tumor injection. Pretreatment with a single dose of 250 μg/mouse of αPD-L1 (BioXcell, West Lebanon, NH) was performed on D41 after tumor inoculation. All injections were i.p. All mice were monitored for survival and were euthanized when showing signs of distress or significant ascites.

### Nanostring analysis

Tumor-bearing mice (D35) were injected with either 4H1128ζ or 4H1128ζ-IL12 CAR T cells. After 48 hr, the animals were euthanized and CD3+ CAR+ or F4/80+ CD11b+ cells were flow sorted and immediately lysed with Trizol (Thermo Fisher scientific) as per manufacturer’s instructions. Collected cells from 2 mice were pooled for each experiment. RNA was purified using a column-based Ambion RNA extraction kit as per manufacturer’s instruction (Life Technologies, Carlsbad, CA, USA). RNA expression levels of 770 genes was detected using nanoString array (nCounter, Gene expression code set, PanCancer Immune Profiling Panel) and analyzed using nSolver Analysis system, PanCancer Pathways Analysis Module (v1.0.48). Normalization of the data was done using the geNorm algorithm automatically performed by the software. Fold change values showing differential expression of 4H1128ζ-IL12 over 4H1128ζ conditions are shown. Data shown with p-values associated with fold changes were derived using the Benjamini-Yekutieli FDR method and set to a threshold of p < 0.05 for significance. Gene expression data have been deposited in National Center for Biotechnology Information’s Gene Expression Omnibus database and are accessible through Gene Expression Omnibus Series accession number GSE97665.

### Statistical analysis

Survival curves were analyzed using Mantel–Cox (log-rank) test and other analysis were performed using unpaired two-tailed T test (p value < 0.05 considered as significant). All calculations were performed using Prism 7 (GraphPad) software.

### Study Approval

All murine studies were done in accordance with Memorial Sloan Kettering Cancer Center Institutional Animal Care and Use Committee approved protocol(00-05-065). All experiments were performed in accordance with relevant guidelines and regulations.

### Data Availability

All data generated and analyzed during this study are included in the published article and supplementary information. Mouse models and antibodies used are commercially available. Cell lines available upon request. Gene expression data have been deposited in National Center for Biotechnology Information’s Gene Expression Omnibus database and are accessible through Gene Expression Omnibus Series accession number GSE97665.

## Electronic supplementary material

Supplementary figures 1-4
