# Supplementary material for: Armored CAR T cells enhance antitumor efficacy and overcome the tumor microenvironment
Source: Sci Rep. 2017 Sep 5;7:10541. doi: 10.1038/s41598-017-10940-8 (PMC5585170; doi:10.1038/s41598-017-10940-8)
Supplement: Supplementary file 1 — Supplementary figures 1-4 [file 41598_2017_10940_MOESM1_ESM.pdf]

Armored CAR T cells enhance antitumor efficacy and overcome the tumor microenvironment

Oladapo O. Yeku[1], Terence J. Purdon[1], Mythili Koneru, David Spriggs[1] and Renier J. Brentjens[1,*]

[1]Department of Medicine, Memorial Sloan Kettering Cancer Center, New York, New York 10065, USA.

*Correspondence to: Renier J. Brentjens; Email: brentjer@mskcc.org

Supplementary figure 1. Transcriptome differences and functional assay on TAMs recovered from animals treated with 4H1128ζ-IL12 and 4H1128ζ CAR T cells. (a). Heatmap representations of differentially expressed genes from pooled recovered TAMs from 4H1128ζ-IL12 and 4H1128ζ- treated mice. **(b).** MHC-II expression on recovered TAMs 48 hr after treatment with 4H1128ζ-IL12 or 4H1128ζ T cells. **(c).** Intracellular and secreted IL-6 and IL-10 levels from TAMs recovered from mice treated with either 4H1128ζ-IL12 and 4H1128ζ T cells. *$p < 0.05$. Data are plotted as mean ± SEM. **(d)**. Arginase activity in recovered TAMs from 4H1128ζ-IL12 and 4H1128ζ- treated mice. Data are plotted as mean ± SEM.

Supplementary figure 2. Endogenous effectors are not required for IL-12 armored CAR T cell efficacy. (a). Female C57BL/6 Ly5.1 mice between 6-8 weeks old were injected i.p with $1x10^7$ ID8-$Muc16^{ecto}$ cells and treated with 4H1128ζ or 4H1128ζ-IL12 CAR T cells derived from C57BL/6 Thy1.2 splenocytes. 48 hr after CAR T cell infusion, peritoneal washes were performed and stained for endogenous Ly5.1 T cells. Recovered Ly5.1 T cells were gated on the $Muc16^-$ $F4/80^-$ population of peritoneal cells, *$p = 0.04$. Data are plotted as mean ± SEM. Data shown are representative results from 3 independent experiments. **(b)**. Female IFN-γ knockout (IFN-$\gamma^{-/-}$) or CD8 knockout ($CD8^{-/-}$) mice between 6-8 weeks old were treated with $2x10^6$ 4H1128ζ or 4H1128ζ-IL12 CAR T cells derived from WT splenocytes 35 days after i.p tumor inoculation, *$p < 0.001$, #$p = 0.001$. n= 5 mice per group. Statistical analysis performed using a log-rank (Mantel-Cox) test.

Supplementary figure 3. Ascites-derived IFN-γ induces upregulation of PD-L1 on ID8-Muc16^{ecto} cells. ID8-Muc16^{ecto} cells cultured for 16 hr in the presence of PBS, pooled ascites with undetectable IFN-γ or pooled ascites with IFN-γ. IFN-γ levels from PBS and asities also shown.

Supplementary figure 4. 4H1128ζ-IL12 CAR T does not lead to increased toxicity in mice. (a). Serum cytokine levels obtained from tumor-bearing WT mice pre- and 7 days post infusion of 1928ζ**,** 1928ζ-IL12, 4H1128ζ and 4H1128ζ-IL12 T cells. $^*p < 0.05$. Data are plotted as mean ± SEM. **(b)**. Non-tumor (NT) and tumor (T) bearing mice were treated with $2x10^6$ i.v 4H1128ζ-IL12 T cells on D0 and $2x10^6$ i.p 4H1128ζ-IL12 T cells on D1 (i.v/i.p) and subsequently weighed. **(c)**. Following i.v/i.p infusion of 4H1128ζ-IL12, blood chemistries, hematologic, liver function and renal function parameters were measured at day 3 and day 16. $^*p < 0.05$, lymphocytes $^*p < 0.05$. Data are plotted as mean ± SEM.

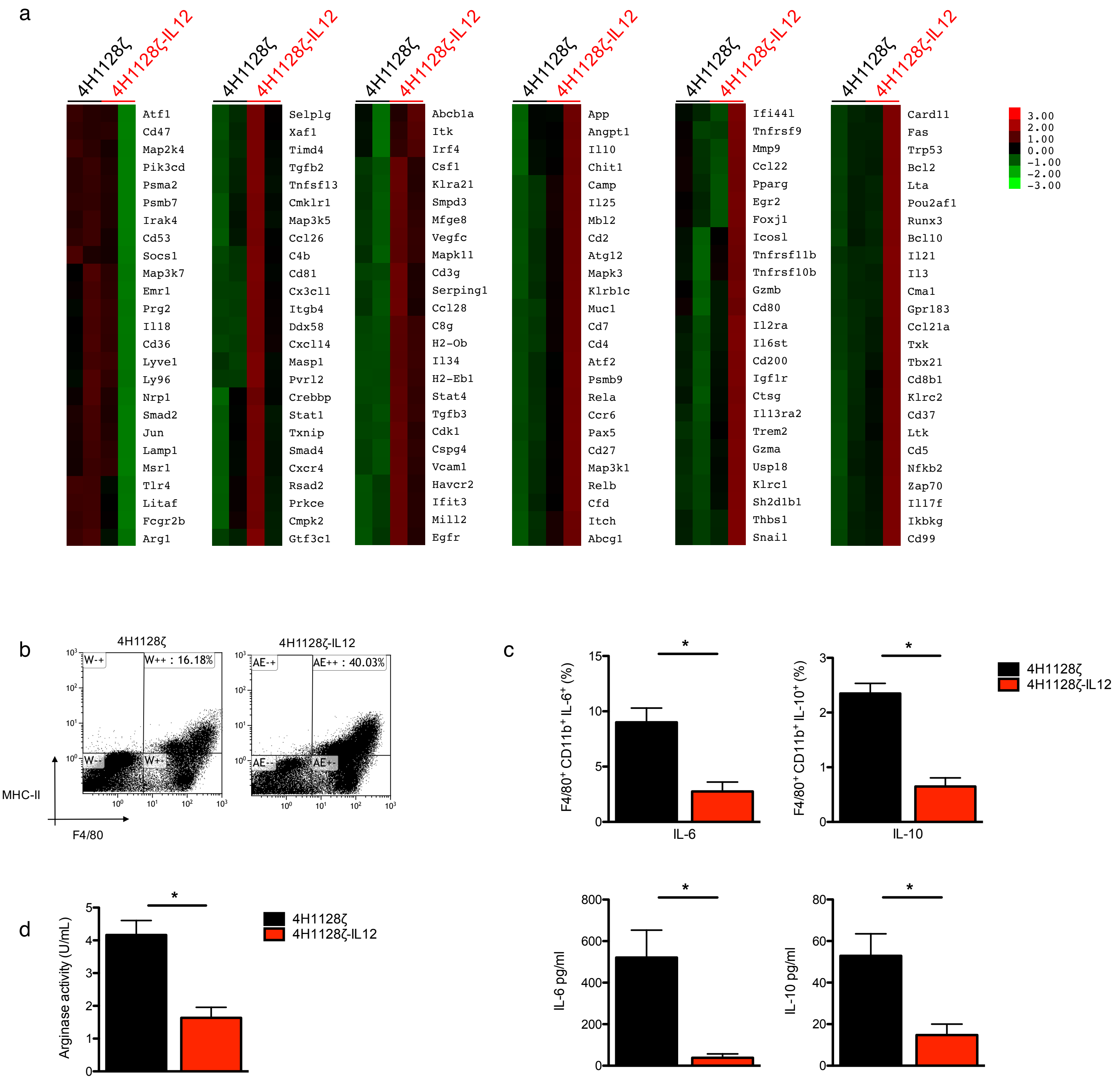
a
4H1128ζ
4H1128ζ-IL12
Atf1 Cd47 Map2k4 Pik3cd Psma2 Psmb7 Irak4 Cd53 Socs1 Map3k7 Emr1 Prg2 Il18 Cd36 Lyve1 Ly96 Nrp1 Smad2 Jun Lamp1 Msr1 Tlr4 Litaf Fcgr2b Arg1
Selplg Xaf1 Timd4 Tgfb2 Tnfsf13 Cmklr1 Map3k5 Ccl26 C4b Cd81 Cx3cl1 Itgb4 Ddx58 Cxcl14 Masp1 Pvrl2 Crebbp Stat1 Txnip Smad4 Cxcr4 Rsad2 Prkce Cmpk2 Gtf3c1
Abcb1a Itk Irf4 Csf1 Klra21 Smpd3 Mfge8 Vegfc Mapk11 Cd3g Serping1 Ccl28 C8g H2-Ob Il34 H2-Eb1 Stat4 Tgfb3 Cdk1 Cspg4 Vcam1 Havcr2 Ifit3 Mill2 Egfr
App Angpt1 Il10 Chit1 Camp Il25 Mbl2 Cd2 Atg12 Mapk3 Klrb1c Muc1 Cd7 Cd4 Atf2 Psmb9 Rela Ccr6 Pax5 Cd27 Map3k1 Relb Cfd Itch Abcg1
Ifi44l Tnfrsf9 Mmp9 Ccl22 Pparg Egr2 Foxj1 Icosl Tnfrsf11b Tnfrsf10b Gzmb Cd80 Il2ra Il6st Cd200 Igf1r Ctsg Il13ra2 Trem2 Gzma Usp18 Klrc1 Sh2d1b1 Thbs1 Snai1
Card11 Fas Trp53 Bcl2 Lta Pou2af1 Runx3 Bcl10 Il21 Il3 Cma1 Gpr183 Ccl21a Txk Tbx21 Cd8b1 Klrc2 Cd37 Ltk Cd5 Nfkb2 Zap70 Il17f Ikbkg Cd99
3.00 2.00 1.00 0.00 -1.00 -2.00 -3.00
b
4H1128ζ
4H1128ζ-IL12
W-+
W++ : 16.18%
W--
W+-
AE-+
AE++ : 40.03%
AE--
AE+-
MHC-II
F4/80
c
F4/80⁺ CD11b⁺ IL-6⁺ (%)
F4/80⁺ CD11b⁺ IL-10⁺ (%)
IL-6
IL-10
IL-6 pg/ml
IL-10 pg/ml
4H1128ζ
4H1128ζ-IL12
d
Arginase activity (U/mL)
4H1128ζ
4H1128ζ-IL12

a

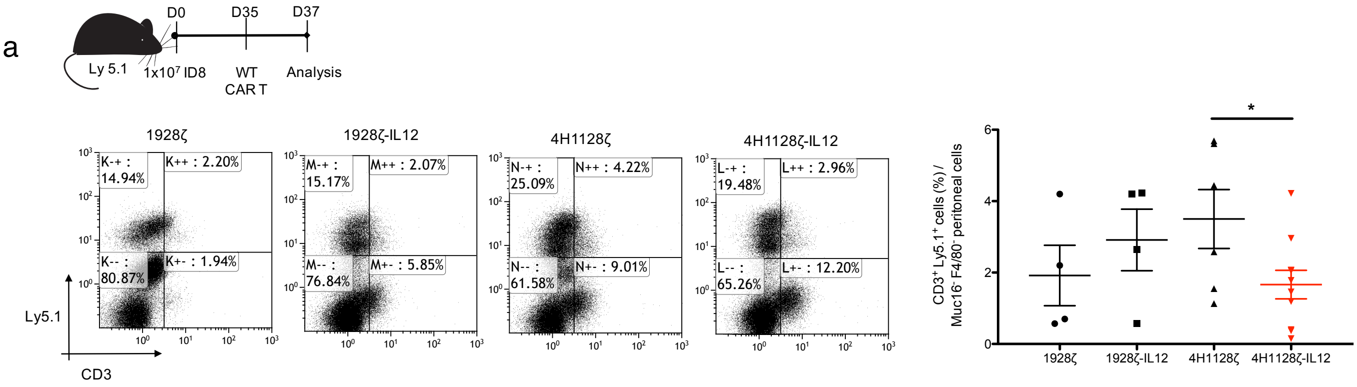

Ly 5.1
1x10^7 ID8
D0
D35
WT CAR T
D37
Analysis
1928ζ
1928ζ-IL12
4H1128ζ
4H1128ζ-IL12
Ly5.1
CD3
CD3+ Ly5.1+ cells (%) / Muc16- F4/80- peritoneal cells

b

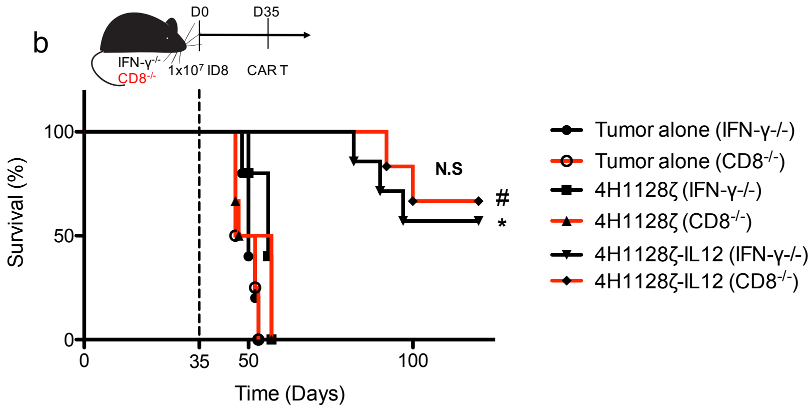

IFN-γ-/-
CD8-/-
1x10^7 ID8
D0
D35
CAR T
Survival (%)
Time (Days)
N.S
Tumor alone (IFN-γ-/-)
Tumor alone (CD8-/-)
4H1128ζ (IFN-γ-/-)
4H1128ζ (CD8-/-)
4H1128ζ-IL12 (IFN-γ-/-)
4H1128ζ-IL12 (CD8-/-)

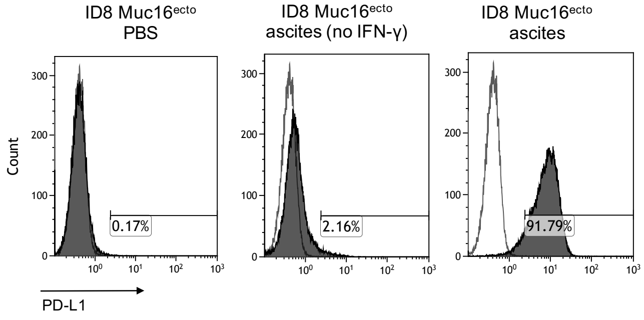

ID8 Muc16ecto PBS
ID8 Muc16ecto ascites (no IFN-γ)
ID8 Muc16ecto ascites
Count
0.17%
2.16%
91.79%
PD-L1

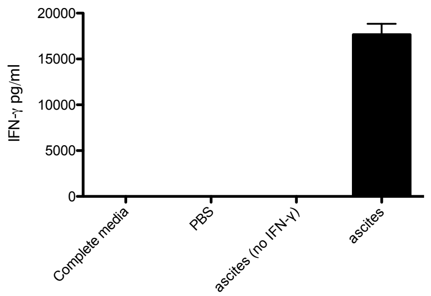

IFN-γ pg/ml
20000
15000
10000
5000
0
Complete media
PBS
ascites (no IFN-γ)
ascites

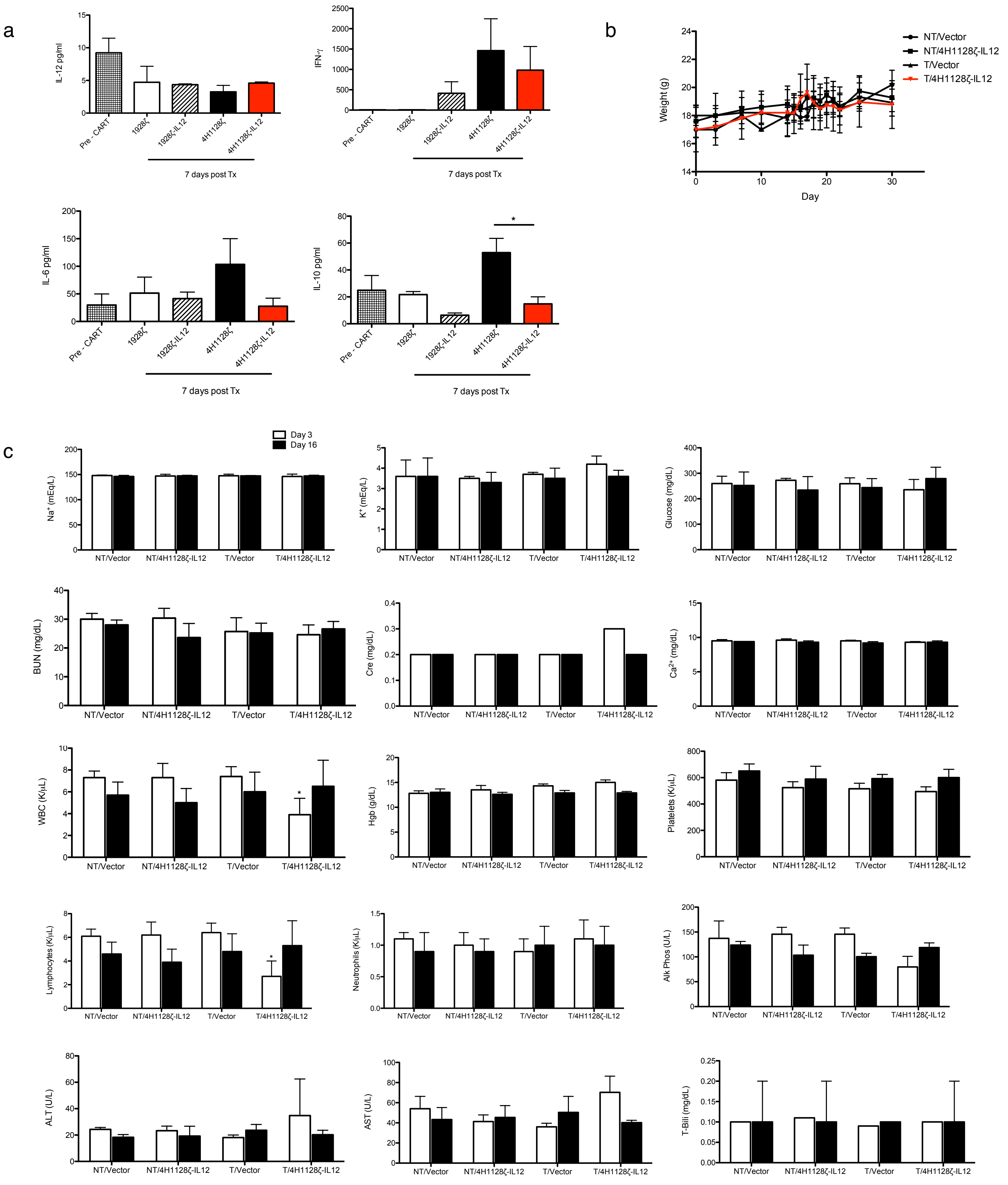
a
IL-12 pg/ml
Pre - CART
1928ζ
1928ζ-IL12
4H1128ζ
4H1128ζ-IL12
7 days post Tx
IFN-γ
IL-6 pg/ml
IL-10 pg/ml
*
b
Weight (g)
Day
NT/Vector
NT/4H1128ζ-IL12
T/Vector
T/4H1128ζ-IL12
c
Day 3
Day 16
Na+ (mEq/L)
K+ (mEq/L)
Glucose (mg/dL)
BUN (mg/dL)
Cre (mg/dL)
Ca2+ (mg/dL)
WBC (K/μL)
Hgb (g/dL)
Platelets (K/μL)
Lymphocytes (K/μL)
Neutrophils (K/μL)
Alk Phos (U/L)
ALT (U/L)
AST (U/L)
T-Bili (mg/dL)
